# Immunohistochemical analysis of cyclin A expression in Wilms tumor

**DOI:** 10.7717/peerj.6212

**Published:** 2019-01-11

**Authors:** Sanja Radojević-Škodrić, Dimitrije Brašanac, Slaviša M. Đuričić, Sofija Glumac, Zlatibor Lončar, Ivan Pavlović, Ana Todorović, Gorana Nikolić, Ivana Baralić, Snežana Pejić

**Affiliations:** 1 Institute of Pathology, School of Medicine, University of Belgrade, Belgrade, Serbia; 2 Department of Clinical Pathology, Mother and Child Health Care Institute of Serbia “Dr. Vukan Čupić”, Belgrade, Serbia; 3 School of Medicine, Banjaluka University, Banjaluka, Bosnia and Herzegovina; 4 Clinic for Emergency Surgery, Clinical Center of Serbia, Belgrade, Serbia; 5 Laboratory of Molecular Biology and Endocrinology, Vinča Institute of Nuclear Sciences, University of Belgrade, Belgrade, Serbia; 6 Department of Biomedical Engineering, Innovation Center, Faculty of Mechanical Engineering, University of Belgrade, Belgrade, Serbia; 7 Zvezdara University Medical Center, Belgrade, Serbia

**Keywords:** Wilms tumor, Immunohistochemistry, Survival, Cyclin A, Retrospective study

## Abstract

**Background:**

Cyclin A overexpression is found in a variety of human tumors and correlates with unfavorable outcome. We analyzed immunohistochemical expression of cyclin A in Wilms tumor (WT) in relation to clinicopathological characteristics, preoperative chemotherapy (PrOpChTh), and overall survival (OS).

**Methods:**

This retrospective study involved 43 patients who underwent nephrectomy from January 1996 to October 2010. Tumor stage and histological subtype were determined by revised Societé International d’Oncologie Pediatrique protocol, based on histological components/alterations caused by PrOpChTh, within the prognostic group of low, intermediate and high risk, and with criteria for anaplasia. The regressive/necrotic changes in total tumor mass of primary tumor and the proportion of epithelial, blastemal, and stromal components in the remaining viable tumor tissue were also determined. Cyclin A expression was evaluated by immunohistochemistry using a polyclonal rabbit, antihuman antibody (H-432).

**Results:**

Cyclin A overexpression was found in 34.3% of WTs, with higher frequency in tumors with epithelial (31.3%) and blastemal (37.1%) components than those with stromal component (17.7%). Regarding histological type, cyclin A overexpression was found most often in focal anaplasia (100%), stromal (60%), and diffuse anaplastic (66.7) WTs. The overexpression was also more frequent in stages 3 and 4 (77.8% and 66.7%, respectively) compared to tumors in stages 1 and 2 (13.3% and 12.5%, respectively; *p* = 0.004) in all components, as well as in blastemal component in stages 3 and 4 (77.8% and 66.7%, respectively) vs. stages 1 and 2 (13.3% and 25%, respectively, *p* = 0.009). Cyclin A overexpression in all components was 66.7% in WTs with metastasis and 31.3% in WTs without metastasis (*p* = 0.265, Fisher test). Log-rank testing revealed differences of OS regarding stage (*p* = 0.000), prognostic groups (*p* = 0.001), and cyclin A expression in blastemal component (*p* = 0.025). After univariate analysis, tumor stage (*p* = 0.001), prognostic group (*p* = 0.004), and cyclin A expression in blastemal component (*p* = 0.042) were significant prognostic factors for OS; however, after multivariate analysis, none of these factors were confirmed as independent predictors of survival.

**Discussion:**

This study showed that cyclin A overexpression might be associated with the development and progression of WT with anaplasia. Also, cyclin A overexpression was more often observed in advanced stages (3 and 4) of WT, in the group of high-risk WTs, and in focal and diffuse anaplasia WTs. There was no relation of cyclin A overexpression and metastatic ability of WT. Although this study has not confirmed the prognostic value of cyclin A overexpression, its association with unfavorable prognosis should be further evaluated.

## Introduction

The cell cycle is regulated by the three main groups of proteins: cyclins, cyclin-dependent kinases (CDKs), and cyclin-dependent kinase inhibitors (CKIs). The CDKs are a family of serine/threonine kinases involved in transitions between phases of cell cycle and they require association with cyclins for their activity (Lim & Kaldis, 2013). Cyclin A is associated with CDK1 and CDK2 and has functions in both S phase and mitosis (Yam, Fung & Poon, 2002). It is expressed from the late G1 phase till early mitosis, reaches a maximum in late S phase and G2 phase (Krabbe, Margulis & Lotan, 2016), when, associating with CDK1, it triggers the initiation of chromosome condensation and possibly nuclear envelope breakdown (Gong et al., 2007). Abnormalities in cell-cycle control underlie the aberrant cell proliferation that characterizes cancer (Williams & Stoeber, 2012), thus immunohistochemical (IHC) assessment of cell-cycle proteins has a diagnostic utility in histopathology (Thway et al., 2012). Association between cyclin A overexpression and poor prognosis was confirmed in a variety of human tumors (Handa et al., 1999; Ito et al., 2002; Chen et al., 2003; Saarilahti et al., 2003). In medulloblastoma, the cyclin A index >40% was associated with poorer survival (Moschovi et al., 2011). In contrast, there are few studies reporting cyclin A overexpression in normal colon mucosa (Wang et al., 1996) and in ovarian carcinomas with a more prolonged survival (Davidson et al., 2001).

Wilms tumor (WT) is one of the most common genitourinary malignant solid tumors in childhood (Radojevic-Skodric et al., 2012). It comprises 90% of pediatric renal tumors (Brok et al., 2016) with an annual incidence of 1 in 10,000 children worldwide (Breslow et al., 1993). The highest incidence of WT occurs in first 4 years of life, and it’s relatively uncommon in children older than 6 years (Martignoni et al., 2007). The WT is genetically heterogeneous disease in which somatic changes include mutations in *Wilms tumor 1 (WT1)* (10–20%), *catenin beta 1 (CTNNB1)* (15%), *Wilms tumor gene on the X chromosome protein* (*WTX)* (15–20%), *tumor protein P53* (*TP53)* (70% of anaplastic tumors) (Park et al., 1993; Koesters et al., 1999; Rivera et al., 2007; Maschietto et al., 2014), and *imprinting control region* that controls expression of *insulin-like growth factor 2* gene and adjacent *H19* (37% of tumors) (Scott et al., 2012). Normal *WT1* expression is essential for blastemal cells differentiation into the mature epithelial component of kidney parenchyma, and disruption of this expression may result in the development of cells with the potential for tumor formation (Grubb et al., 1994). Latest research has shown that gain of 1q is associated with poorer survival in WT patients in addition to histologic response to preoperative chemotherapy (PrOpChTh) and tumor stage (Chagtai et al., 2016). Genomic gain of the *N-Myc proto-oncogene protein (MYCN)* is associated with poor prognosis in several childhood cancers. Some results suggest a significant role for *MYCN* dysregulation in the molecular biology of WT and conclude that *MYCN* gain is prognostically significant for this type of disease (Williams et al., 2015). However, the only genomic biomarker associated with poor outcome is simultaneous loss of heterozygosity of chromosomes 1p and 16q, and it was validated in WT patients with surgery as the first therapeutic intervention (Grundy et al., 2005; Gratias et al., 2016). Some studies regarding IHC examination of cell cycle regulators in WT have been published so far. Different authors have shown that antigen Ki67 proliferation index was significantly correlated with tumor stage in WT patients (Krishna et al., 2016) but also that blastemal type of WT shows more pronounced expression of CKI 2A (p16) and cyclin E (Radojevic-Skodric et al., 2007; Basta-Jovanovic et al., 2008). The higher values of cyclin E were also observed in WT with metastases and recurrences (Taran et al., 2011). Expression of tumor markers WT1, transforming growth factor alpha, vascular endothelial growth factor, E3 ubiquitin-protein ligase (MIB1), and CKI 1B (p27Kip1) correlates with clinical progression of tumor (Ghanem et al., 2013), while Ki67 is a relevant marker for assessing the proliferative activity, but it may not be a good clinical prognostic marker (Jurić et al., 2010).

The aim of this study was to analyze cyclin A IHC expression in WT metastases and primary tumors, as well as its relation to clinicopathological characteristics and overall survival (OS). We hypothesized that cyclin A overexpression may be associated with histopathological finding of WT and progression.

## Materials and Methods

### Study design and patients

This retrospective study was conducted on tumor specimens and clinical data of patients with WT who underwent nephrectomy between January 1996 and October 2010. All specimens were obtained from the archives of Institute of Pathology, School of Medicine, University of Belgrade, Serbia (where the study was performed) and Mother and Child Health Care Institute of Serbia “Dr. Vukan Čupić,” Belgrade, Serbia. Of the available 59 samples, fourteen cases were excluded due to inadequate sampling or incomplete clinical data and two were excluded as bilateral tumors thus, the study was limited to 43 patients.

For each patient included in the study, available clinical–morphological data were collected by reviewing the patients’ medical histories. The data collected included sex, age (months), tumor stage, histological type, and prognostic group (Table 1). The mean age of patients was 52.4 months (range 7–132 months) and there were 28 (65.1%) females and 15 (34.9%) males. The histological diagnoses and classification were performed in accordance with the revised Societé International d’Oncologie Pediatrique (SIOP) Working Classification (Vujanić et al., 2002) criteria for histological subtypes (according to Section A) and strict adherence to the criteria for anaplasia (Vujanić et al., 1999). Based on the histological material, WTs were classified into following risk groups: (a) low risk (cystic partially differentiated WT), (b) intermediate risk (IR; regressive, epithelial, stromal, or mixed), and (c) high risk (blastemal or diffuse anaplasia) (Vujanić et al., 2002). Examination of prognostic significance of histologic components of WT characterized by a different degree of aggressiveness, but also different sensitivity to chemotherapy, provides a more precise classification of tumor subtypes within a group of medium risk and hence the adaptation of therapeutic protocols.

**Table 1:** Clinical data, morphological features and cyclin A expression in primary Wilms tumors and metastasis.

Number	Age (months)	Sex	Preoperative therapy	Tumor size (cm)	Stage	Histological type	Prognostic group	Cyclin A expression in tumor components			
								E	B	S	T
1	23	F	Yes	7	I	Mixed	IR	+	+	+	+
2	14	F	Yes	8	II	Stromal	IR	++	++	+	++
3*	18	F	Yes	7	IV	Stromal	IR	+	++	++	++
4	24	F	Yes	7	I	Stromal	IR	+	+	+	+
5	48	F	Yes	6	I	Mixed	IR	−	−	−	−
6	36	F	Yes	6	I	Blastemal	HR	−	+	+	+
7	60	F	Yes	6	I	Blastemal	HR	+	+	+	+
8	24	M	Yes	8	III	Anaplastic	IR	+	+	+	+
9	84	M	Yes	8	III	Anaplastic	HR	++	++	++	++
10	132	M	Yes	12	I	Blastemal	HR	−	−	−	−
11	36	F	Yes	7	II	Blastemal	HR	+	+	+	+
12	84	M	Yes	13	I	Blastemal	HR	+	+	+	+
13	24	F	Yes	10	I	Epithelial	IR	+	+	+	+
14	12	F	Yes	10	I	Epithelial	IR	++	+	/	+
15	96	F	Yes	16	III	Stromal	IR	−	++	++	++
16	132	F	Yes	19	III	Anaplastic	HR	++	++	+	++
17	24	F	Yes	6	I	Stromal	IR	+	+	+	+
18	57	M	Yes	8	II	Blastemal	HR	+	++	+	+
19	79	M	Yes	5	II	Mixed	IR	+	+	+	+
20	132	F	Yes	4	III	Regressive	IR	−	−	−	−
21*	36	F	Yes	3	IV	Regressive	IR	++	+	+	+
22	24	F	Yes	6	II	Regressive	IR	+	+	++	+
23	36	F	Yes	12	I	Mixed	IR	++	++	++	++
24	60	M	Yes	9	II	Regressive	IR	+	+	+	+
25	24	M	Yes	5	III	Mixed	IR	/	++	+	++
26	132	F	Yes	8	III	Anaplasia	IR	++	++	+	++
27	36	F	Yes	12	I	Mixed	IR	/	+	+	+
28	108	F	Yes	5	II	Regressive	IR	+	−	−	+
29	72	F	Yes	7	I	Mixed	IR	+	+	+	+
30*	60	F	Yes	10	IV	Blastemal	HR	/	++	+	++
31	24	F	Yes	14	III	Mixed	IR	++	++	+	++
32	48	F	Yes	9	II	Regressive	IR	−	−	−	−
33	24	M	Yes	10	III	Regressive	IR	++	++	+	++
34	60	M	Yes	9	I	Anaplasia	HR	++	++	+	++
35	24	M	Yes	7	I	Mixed	IR	+	+	+	+
36	7	M	No	7	I	Blastemal	IR	+	+	+	+
37	43	F	No	9	I	Blastemal	IR	−	+	−	+
38	55	M	No	8	I	Anaplastic	HR	++	++	+	++
39*	25	F	No	7	IV	Blastemal	IR	++	++	++	++
40	64	F	No	3	I	Blastemal	IR	++	+	+	+
41	16	F	No	5	I	Mixed	IR	+	++	+	+
42	79	M	No	9	I	Blastemal	IR	++	++	+++	++
43	55	M	No	16	I	Anaplastic	HR	/	+	+	+
m3								+++	++	++	++
m21								+++	/	+++	++
m30/1								++	/	++	++
m30/2								++	/	++	++
m39								++	++	++	++

**Notes:**

M, male; F, female; IR, intermediate risk; HR, high risk; E, epithelial component; B, blastemal component; S, stromal component; T, all components; −, no staining; +, staining in less than 10% of cells; ++, staining in 10–50% of cells; +++, staining in more than 50% of cells; /, absence of epithelial, blastemal, or stromal component; m, metastasis.

* Primary tumor with pulmonary metastasis.

A total of 35 (81.4%) patients were treated according to SIOP protocols that include PrOpChTh for all patients older than 6 months, surgical operation, postoperative chemotherapy, and in some cases radiation therapy, as well as other medical standards and procedures in diagnosing and treating diseases (SIOP 9, SIOP 6, and some according to 93-01, depending on the period when the diagnosis was established). For each primary tumor, the percentage of regressive/necrotic changes in the total tumor mass was determined by a semiquantitative method, based on the macroscopic and microscopic examination. In the remaining viable tumor tissue, the proportion of epithelial, blastemal, and stromal components was determined microscopically. Seven (20%) cases were predominantly blastemal histological type, nine (25.7%) mixed, seven (20%) regressive, five (14.3%) stromal, and two (5.7%) predominantly epithelial. Among anaplastic tumors, three (8.6%) were diffusely anaplastic and two (5.7%) showed focal anaplasia. The revised SIOP staging criteria (Vujanić et al., 2002) was also used to determine tumor stage. Stage 1 was diagnosed in 15 (42.8%) WT cases, stage 2 in eight (22.8%), stage 3 in nine (25.7%), and stage 4 in three (8.6%) cases. According to the prognostic group, 25 (71.4%) cases were classified as intermediate (IR) group and 10 (28.6%) cases as high-risk group (HR).

Eight patients (18.6%) were not treated with PrOpChTh and they were analyzed separately. These cases were classified according to the Children’s Oncology Group criteria (blastemal type–IR, and not the high risk as it would be in SIOP). Five cases (62.5%) were predominantly blastemal histological type, 2 diffusely anaplastic (25%) and 1 (12.5%) was mixed histological type.

The follow-up period was 5 years and the OS time was defined as a period between date of surgery and date of death. Patients still alive at the end of the study were censored. Since according to the State Laws and Legislation patients are not required to receive therapy or to be followed up in the same clinic where they were diagnosed and operated, the event free survival could not be analyzed due to insufficient number of subjects to be included in the analysis.

The study was conducted in accordance with the ethical standards of the 1964 Helsinki Declaration and applicable national regulations. The ethical approval was received from Ethics Committee of School of Medicine, University of Belgrade, (1600/I-38). For this type of study formal consent is not required.

### Methods

**Figure 1:**
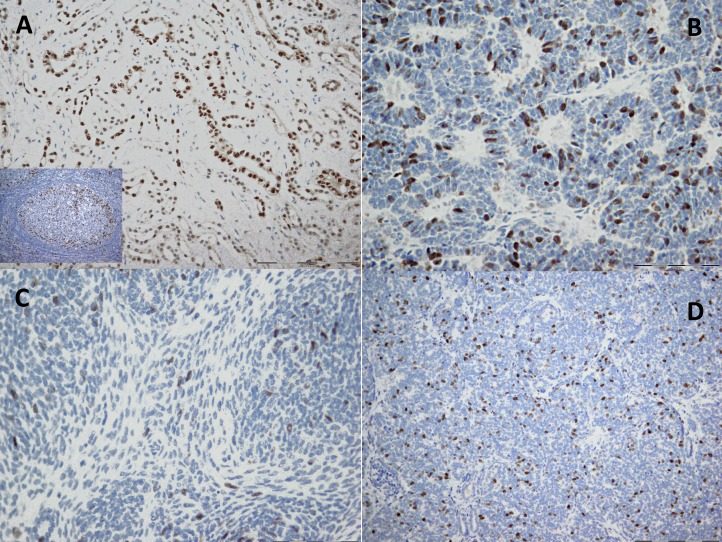
Immunohistochemical staining of cyclin A in kidney tissue. (A) Normal tissue where cyclin A shows focal nuclear and cytoplasmic staining in tubular epithelial cells (20×). (B–D) Tumor where cyclin A shows diffuse nuclear staining in: (B) epithelial component (40×), (C) stromal component (40×) and (D) blastemal component (20×). The IHC staining of normal lymph node tissue was used as a positive control (the image is included in (A)).

For each tumor, at least two paraffin-embedded tissue sections, representative of the global histology, were selected for IHC analysis of cyclin A. Tissue samples from formalin-fixed paraffin blocks and polyclonal rabbit antihuman antibody (H-432; Santa Cruz Biotechnology, Inc., Santa Cruz, CA, USA) were used. Deparaffinized samples were treated 15 min with hydrogen peroxide solution (3%) to stop peroxidase activity. Thereafter, samples immersed in 10 mM citrate buffer (pH 6.0) were treated in a microwave oven (20 min, 620 W) for antigen retrieval. Nonspecific staining was prevented with blocking peptide (DAKO, Glostrup, Denmark) the primary antibody for cyclin A (diluted 1:200) was applied and left overnight at 4 °C. Detection was done with streptavidin-biotin technique (Dako REALTM Detection Systems LSAB™+; DAKO, Glostrup, Denmark). Finally, chromogen (diaminobenzidine) was applied for IHC development and Mayer’s hematoxylin for the counterstaining. IHC stained normal lymph node tissue was used as a positive control, whereas negative control was based on the omission of primary antibody. Strong nuclear staining was considered positive for cyclin A1. The IHC expression of cyclin A was scored with semiquantitative technique (Table 1) as previously described (Todorovic et al., 2014). In normal kidney parenchyma adjacent to the tumor, focal expression of cyclin A was found in epithelial cells of distal convoluted tubules (Figs. 1A–1D) while the expression was absent in glomerular cells.

Consequently, moderate and diffuse expression in WT was considered as cyclin A overexpression. For statistical analyses, cases with no or focal expression and those with moderate or diffuse expression were grouped together, respectively.

### Statistical analysis

Statistical analyses were performed at IC Faculty of Mechanical Engineering, Belgrade, Serbia, with SPSS 23.0 for Windows (SPSS Inc., Chicago, IL, USA) and GraphPad Prism 4. Normality of distribution was tested with Shapiro–Wilks test. For testing the differences between parameters the Pearson χ^2^ test, Fisher exact test and t-test were used. The OS was calculated by Kaplan–Meier method and groups were compared using Log-rank statistics. Univariate and multivariate Cox regression were used to analyze the independent factors related to OS. The *p* < 0.05 was considered significant.

## Results

### Cyclin A expression in all components of WT

The overexpression of cyclin A (Table 2) was almost equal in preoperatively nontreated and PrOpChTh WTs (37.5% vs. 34.3%, respectively, *p* > 0.05, Fisher test).

**Table 2:** The frequency of cyclin A overexpression in different histological types of Wilms tumor (A) patients who received preoperative therapy and (B) patients who did not receive preoperative therapy.

Histological type	Cyclin A overexpression (++/+++)			
	E	B	S	T
**A**				
Epithelial	1/2 (50%)	0/2 (0%)	0/1 (0%)	0/2 (0%)
Stromal	1/5 (20%)	3/5 (60%)	2/5 (40%)	3/5 (60%)
Mixed	2/7 (28.6%)	3/9 (33.3)	1/9 (11.1%)	3/9 (33.3%)
Regressive	2/7 (28.6%)	1/7 (14.3%)	1/7 (14.3%)	1/7 (14.3%)
Focal anaplasia	2/2 (100%)	2/2 (100%)	0/2 (0%)	2/2 (100%)
Blastemal	0/6 (0%)	2/7 (28.6%)	1/7 (0%)	1/7 (14.3%)
Diffuse anaplastic	2/3 (66.7%)	2/3 (66.7%)	1/3 (33.3%)	2/3 (66.7%)
**B**				
Mixed	0/1 (0%)	1/1 (100%)	0/1 (0%)	0/1 (0%)
Blastemal	3/5 (60%)	2/5 (40%)	2/5 (40%)	2/5 (40%)
Diffuse anaplastic	1/1 (100%)	1/2 (50%)	0/2 (0%)	1/2 (50%)

**Note:**

E, epithelial component; B, blastemal component; S, stromal component; T, all components; ++/+++, positive staining in 10–50% of cells and in more than 50% of cells, respectively.

In patients with PrOpChTh, tumors with cyclin A overexpression were larger (10.5 ± 4.06 cm) than those with low cyclin A level (7.52 ± 5.57 cm), (*p* = 0.012, *t*-test). Cyclin A overexpression was found more often in stages 3 and 4 (77.8%, 66.7%, respectively) than in stages 1 and 2 (13.3% and 12.5%, respectively), (*p* = 0.004, Pearson χ^2^), (Table 1). Regarding histological type, cyclin A overexpression was found most often in focal anaplasia, stromal, and diffuse anaplastic WTs (Table 2). No difference was found between diffuse anaplastic and other histological types (*p* = 0.266, Fisher test). In 8/25 (32%) cases of IR tumors and in 4/10 (40%) cases of HR tumors we detected cyclin A overexpression, but without difference (*p* = 0.706, Fisher test), (Table 1).

### Cyclin A expression in individual components of WT

Epithelial and blastemal components of all WTs examined, overexpressed cyclin A with similar frequency (31.3% and 37.1%, respectively; *p* = 0.797), and more often than the stromal component (17.7%, *p* = 0.255 and *p* = 0.106, respectively; Fisher test), (Table 2). Epithelial component was not present in three cases. Similar to analysis of all components together, in epithelial component, we found no difference in cyclin A overexpression in relation to PrOpChTh (*p* = 0.225), tumor size (*p* = 0.088), and prognostic category (*p* = 1.000, Fisher test). Cyclin A overexpression was detected in 55.6% and 33.3% in stages 3 and 4, respectively and in 20% and 12.5% in stages 1 and 2, respectively (*p* = 0.187) (Table 1). There was no difference in cyclin A overexpression between diffuse anaplastic and other histological types (*p* = 0.224, Fisher test). In blastemal component, the cyclin A overexpression was more frequent in stages 3 and 4 (77.8% and 66.7%, respectively) compared to stages 1and 2 (13.3% and 25%, respectively, *p* = 0.009, Pearson χ^2^), (Table 1), whereas there was no difference in relation to PrOpChTh (*p* = 0.691), prognostic category (*p* = 0.444), and tumor size (*p* = 0.164, Fisher test). Overexpression of cyclin A related to histological type showed no difference between diffuse anaplastic and other histological types (*p* = 0.541, Fisher test), (Table 2). The stromal component was not present in one sample. The cyclin A overexpression (Table 1) revealed no difference in relation to stages 3 and 4 (22.2% and 66.7%, respectively) and stages 1 and 2 (6.67% and 12.5%, respectively), (*p* = 0.085, Pearson χ^2^). There was no difference in relation to PrOpChTh (*p* = 0.635), tumor size (*p* = 0.148), prognostic category (*p* = 1.000), and between diffuse anaplastic WT and other histological types (*p* = 0.442), (Table 2).

### Cyclin A expression in primary WT and WTs with metastasis

Cyclin A overexpression in all components was more frequent in WTs with metastasis compared to WTs without metastasis (Table 1) but without difference (66.7% vs. 31.3%, *p* = 0.265, Fisher test). There was also no significant difference in frequency of cyclin A overexpression in individual components between WTs with and WTs without metastasis (Table 1). We did not detect cyclin A overexpression in only one case of primary tumor with metastasis, which was regressive histological type.

### Survival analysis

**Figure 2:**
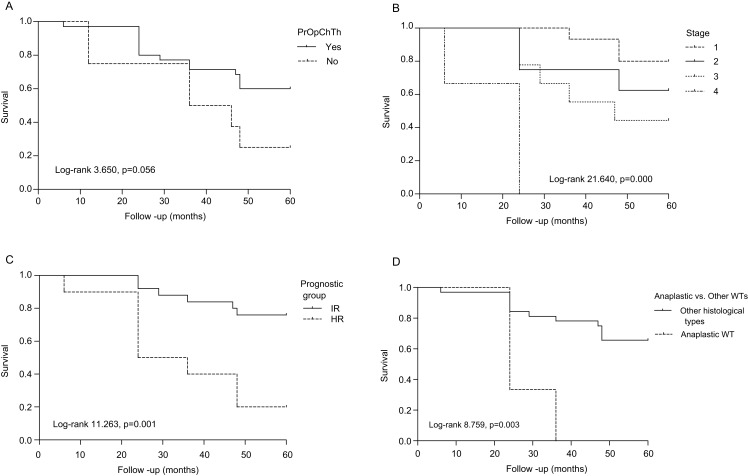
Overall survival of patients with Wilms tumor according to clinicopathological parameters. (A) Preoperative chemotherapy treatment (PrOpChTh), (B) tumor stage, (C) prognostic group: intermediate risk (IR), high risk (HR), and (D) histological type (diffuse anaplastic vs. other types of WT).

**Figure 3:**
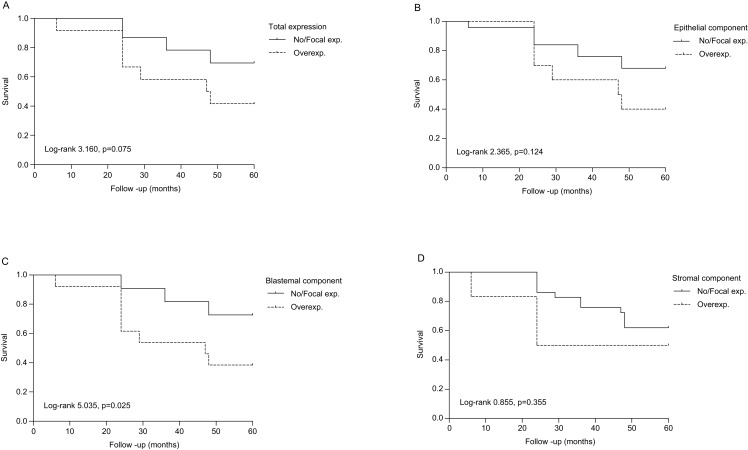
Overall survival of patients with Wilms tumor stratified by cyclin A expression. (A) All components (total expression), (B) epithelial component, (C) blastemal component, and (D) stromal component. No/focal–absence of staining and positive staining in less than 10% of cells, respectively; overexpression–positive staining in 10–50% of cells and in more than 50% of cells, respectively.

The OS analysis (Fig. 2A) revealed that 97.1%, 71.4%, and 60% of PrOpChTh patients and 75%, 50%, and 25% of patients without therapy were alive after 1, 3, and 5 years, respectively (Log-rank 3.650, *p* = 0.056). The correlation was found for survival and stage (Log-rank 21.640, *p* = 0.000), and prognostic group (Log-rank 11.263, *p* = 0.001), (Figs. 2B–2C). After 5 years of follow-up, patients with stage 1 and 2 tumors had OS of 80% and 62.5%, respectively, while patients with stage 3 and 4 had OS of 44.4% and 0%, respectively. The rates of OS after 1, 3, and 5 years for patients with IR prognostic group were 100%, 84%, and 76%, respectively; patients with HR had survival rates of 90%, 40%, and 20% after 1, 3, and 5 years. In the group of patients with diffuse anaplastic WT, the OS was 0% after 3-year period in comparison to all other histological types (Log-rank 8.759, *p* = 0.003), (Fig. 2D). There was no difference in OS between patients related to gender (Log-rank 2.763, *p* = 0.096). In regard to OS and cyclin A expression, there was no correlation in all WT components (total expression), (Log-rank 3.160, *p* = 0.075), while in individual WT components the correlation was found only in blastemal component (Log-rank 5.035, *p* = 0.025). Patients who overexpressed cyclin A in blastemal component had the 5-year OS of 38.5%, (Figs. 3A–3D).

Univariate analysis (Table 3) showed that tumor stage (*p* = 0.001), prognostic group (*p* = 0.004), as well as cyclin A expression in blastemal component (*p* = 0.042), were significant prognostic factors for OS. After multivariate analysis, none of these factors remained statistically significant.

**Table 3:** Factors affecting overall survival in patients with Wilms tumor.

Variable	Univariate analysis			
	Coefficient *b*	HR	95% CI	*p*
Prognostic group				
IR		1.0		
HR	1.581	4.859	1.668–14.152	0.004
Stage				
Stage 1		1.0		
Stage 2	0.796	2.216	0.447–10.995	0.330
Stage 3	1.371	3.938	0.931–16.655	0.062
Stage 4	3.182	24.102	3.879–149.742	0.001
B (cyclin A expression)				
No/focal		1.0		
Overexpression	1.107	3.025	1.043–8.777	0.042

## Discussion

The results of our study showed that cyclin A overexpression in WT was more frequent in advanced stages and tumors with metastasis, which indicated that cyclin A overexpression may be associated with tumor progression. Cyclins play a multifunctional role in cancer pathogenesis and alterations in their structure and function can lead to an array of cancer types (Stamatakos et al., 2010). Prognostic value of cyclins in WT was also investigated previously (Radojevic-Skodric et al., 2007; Basta-Jovanovic et al., 2008). Faussillon et al. (2005) reported increased expression of cyclin D2 in 86% of WT cases. Berrebi et al. (2008) showed that cyclin E overexpression may have prognostic value in WT. Similarly, Taran et al. (2011) observed higher values of cyclin E1 related to WT cases with less favorable prognosis. However, to our knowledge, analysis of cyclin A expression in WT has not been reported. Normal kidney tissue, analyzed in this study, showed focal cyclin A expression. This finding confirms that normal renal tissue has a low proliferative capacity (Clapp & Cloker, 1997). In our study, about 34% WT showed cyclin A overexpression, defined as increased level of cyclin A comparing with normal kidney tissue. Cyclin A overexpression was found in different human tumors in adults (Aaltomaa et al., 1999; Poikonen et al., 2005; Sørby et al., 2012; Cyniak-Magierska et al., 2015), as well as in pediatric embryonal tumors (Moschovi et al., 2011) and in neuroblastoma tumor NI65 (Molenaar et al., 2003). In contrast, Wang et al. (1996) reported decreased expression of cyclin A in colon cancer compared to normal colon mucosa in 63% cases.

The overexpression of cyclin A has also been considered with reference to the sensitivity for chemotherapy. Huuhtanen et al. (1999) reported association between higher cyclin A score and good chemotherapy response in soft tissue sarcoma patients but no such association was found in breast cancer patients (Poikonen et al., 2005). On the other hand, cyclin A overexpression should also be considered in terms of chemotherapy resistance (Cybulski et al., 2015; Huang et al., 2016). In our study, cyclin A overexpression (total, epithelial, blastemal, and stromal component) was not found to be related with PrOpChTh treatment. Survival analysis showed the similar OS rate between untreated patients and those treated with PrOpChTh.

We also investigated the association between cyclin A overexpression and the WT size. Although tumors that showed cyclin A overexpression were larger, no difference proved to be statistically significant. In previous reports on different tumors, cyclin A overexpression demonstrated good correlation with tumor grade (Handa et al., 1999; Ito et al., 2002; Saarilahti et al., 2003; Sørby et al., 2012), but not with tumor size (Oda et al., 2003; Ha et al., 2012). There have been no results regarding cyclin A or any other cyclin expression in relation to size of WT published so far. The expression of cyclin A was more frequent in stages 3 and 4 compared to stages 1 and 2. Other studies also showed that cyclin A overexpression was significantly linked to the advanced stages of different tumors (Chen et al., 2003; Oda et al., 2003; Miao et al., 2015). Conversely, there was no difference in cyclin A expression between different stages of colorectal carcinoma (Handa et al., 1999), laryngeal cancer (Saarilahti et al., 2003), as well as non-small lung cancers (Ha et al., 2012). The stage of WT is well known independent prognostic factor, therefore this statistically significant correlation between the WT stage and cyclin A overexpression suggests that increased expression of cyclin A may be related to the poor prognosis of WT. This is in accordance with our survival analysis. In our cohort, in comparison to stage 1, patients with stages 3 and 4 had about 4 and 24 times greater risk of dying, while those with stage 2 had the similar risk.

We found overexpressed cyclin A more often in epithelial and blastemal component compared to stromal. The most investigated marker of proliferative activity of WT is Ki-67, which is also far more often expressed in epithelial and blastemal than in stromal component of WT (Juszkiewicz et al., 1997; Berrebi et al., 2008). The results of our and these previous studies (Nagoshi & Tsuneyoshi, 1994) could be explained by WT development theory (Beckwith, 1993; Beniers et al., 2001). Namely, normal differentiation of blastemal component, which arises from metanephric blastema, is disrupted and consequently immature tubules and abortive glomerules are formed, with the more pronounced proliferative capacity. There was no significant difference in cyclin A expression between histological types of WT. However, overexpression of cyclin A (total expression and expression in individual components of WT) was observed most often in diffuse anaplastic and focal anaplasia. This result is in agreement with the results of other studies, which showed correlation between overexpression of cyclin A and poorly differentiated tumors (Handa et al., 1999; Ito et al., 2002; Saarilahti et al., 2003).

Although histological features and stage of WT play the most important role in the assessment of prognosis, there is a growing number of molecular biology markers that possibly could allow identification of tumors with worse (HR–WT) and better (IR–WT) prognosis. In our study, we found cyclin A overexpression more often in HR prognostic group (blastemal type of WT after receiving chemotherapy and WTs with diffuse anaplasia) than in IR prognostic group, but the observed difference was not significant. This is mainly due to an absence of association of cyclin A overexpression with blastemal type WT, while tumors with diffuse anaplasia showed a high frequency of elevated cyclin A expression. Blastemal type WT has been included in high-risk category WT in the last SIOP revision of classification of renal tumor of childhood (Vujanić et al., 2002). We found no difference in cyclin A levels in blastemal WT regarding the PrOpChTh usage. Although it is assumed that resistance to chemotherapy is a common denominator, our results could suggest that WT with diffuse anaplasia, in addition, have increased proliferation capacity. In our study, cyclin A overexpression was most often in WT with focal and diffuse anaplasia, and recently it has been suggested that focal anaplasia could have similar prognostic significance as the diffuse (Sebire & Vujanic, 2009). The HR prognostic group in our study had about five times greater risk of dying than the IR group of patients. In our study, cyclin A overexpression was found more often in primary WT tumors with metastasis compared to those without metastasis but without significant difference. Different results were obtained for cyclin E in WT. Berrebi et al. (2008) indicated that cyclin E was correlated with tumor aggressiveness and metastases, and that assessment of its expression may have prognostic value in the categorization of WT.

## Conclusions

Our results do not confirm prognostic significance of cyclin A; however, this study showed that cyclin A overexpression might be associated with the development and progression of WT with anaplasia. Also, cyclin A overexpression was more frequently observed in advanced stages (3 and 4) of WT, in the group of high-risk WTs, and in focal and diffuse anaplasia WTs, which suggest that cyclin A overexpression may be associated with the unfavorable prognosis. Based on our results there was no relation of cyclin A overexpression and metastatic ability of WT. The larger cohort of patients would provide a better evaluation of the association of cyclin A overexpression and unfavorable prognosis in WT patients. The limitations of this study are a small number of patients and retrospective design.

## Additional Information and Declarations

### Data Availability

The following information was supplied regarding data availability: The raw data are provided in Table 1.
